# Pleuroparenchymal Fibroelastosis: A New Entity within the Spectrum of Rare Idiopathic Interstitial Pneumonias

**DOI:** 10.1155/2015/810515

**Published:** 2015-08-26

**Authors:** Karina Portillo, Ignasi Guasch, Caroline Becker, Felipe Andreo, Maria Teresa Fernández-Figueras, José Ramirez Ruz, Carlos Martinez-Barenys, Samuel García-Reina, Pedro Lopez de Castro, Irene Sansano, Ana Villar, Juan Ruiz-Manzano

**Affiliations:** ^1^Department of Pulmonary Medicine, Hospital Universitari Germans Trias i Pujol, Universitat Autónoma de Barcelona, Badalona, 08916 Catalonia, Spain; ^2^Department of Radiology, Hospital Universitari Germans Trias i Pujol, Universitat Autónoma de Barcelona, Badalona, 08916 Catalonia, Spain; ^3^CIBER de Enfermedades Respiratorias (CIBERES), Instituto de Salud Carlos III, Palma de Mallorca, Spain; ^4^Department of Pathology, Hospital Universitari Germans Trias i Pujol, Universitat Autónoma de Barcelona, Badalona, 08916 Catalonia, Spain; ^5^Department of Pathology, Hospital Clínic, University of Barcelona, Barcelona, 08036 Catalonia, Spain; ^6^Department of Thoracic Surgery, Hospital Universitari Germans Trias i Pujol, Universitat Autónoma de Barcelona, Badalona, 08916 Catalonia, Spain; ^7^Pathology Department, Hospital Universitari Vall d'Hebron, Universitat Autónoma de Barcelona, Barcelona, 08035 Catalonia, Spain; ^8^Department of Pulmonary Medicine, Hospital Universitari Vall d'Hebron, Universitat Autónoma de Barcelona, 08035 Catalonia, Spain; ^9^Department of Internal Medicine, Universitat Autónoma de Barcelona, Catalonia, Spain

## Abstract

Pleuroparenchymal fibroelastosis (PPFE) is a rare entity that has been recently included in the official American Thoracic Society/European Respiratory Society (ATS/ERS) statement in 2013 as a group of rare idiopathic interstitial pneumonias (IIPs). PPFE is characterized by pleural and subpleural parenchymal thickening due to elastic fiber proliferation, mainly in the upper lobes. The etiology of the disease is unclear, although some cases have been associated as a complication after bone marrow transplantation, lung transplantation (LT), chemotherapy, and recurrent respiratory infections. The patients usually report progressive dyspnea and dry cough and are predisposed to develop spontaneous or iatrogenic pneumothoraces after surgical lung biopsy (SLB) for its diagnosis. That is why better awareness with the clinical and radiologic features can help optimal management by the multidisciplinary team. Novel invasive techniques such as cryobiopsy may become useful tools in these patients as it could spare SLB. We present the first reported cases in Spain.

## 1. Introduction

Pleuroparenchymal fibroelastosis (PPFE) is a rare condition that is characterized by fibrosis involving the pleura and subpleural lung parenchyma, predominantly in the upper lobes [1, 2]. There is a wide range in age of onset, from young to old, although in most of the published series it is seen in adults in their third and fourth decades of life [3]. There is no gender preponderance [2]. The etiology of the disease is unclear, although association with different conditions such as previous chemotherapy treatment, bone marrow and lung transplantation (LT), and recurrent pulmonary infections has been described [4–8]. The most common reported symptoms are dyspnea, nonproductive cough, weight loss, and pneumothorax. Thoracic cage deformity “platythorax” (due to abnormal narrowing of the ratio of the anterior-posterior diameter of the thorax to the transverse diameter of the thorax) is a common clinical sign and is an indicator of disease progression [3, 6].

We present two cases of young adults. In the first case report, PPFE was not initially suspected. Cryobiopsy was performed but the findings were inconclusive for diagnosis since the appropriate histological staining was not requested. Due to rapid clinical and radiological deterioration evidenced by high resolution computed tomography (HRCT), the suspicion of PPFE was raised leading to pathologists to perform elastic fiber stain (procedure not routinely done) on the surgical lung biopsy (SLB). The patient successfully underwent bilateral lung transplantation (LT) with 24-month survival up to now. The second case report describes a woman with previous chemotherapy and radiotherapy treatment for breast cancer, whose first symptom was spontaneous pneumothorax. Diagnosis was also made by SLB. Both patients presented with apical pneumothorax as a complication of this procedure.

## 2. Case Reports

*Case  1*. A 25-year-old man referred to our hospital with six-month progressive dyspnea, asthenia, and nonproductive cough. His past medical history included childhood multicentric Castleman's disease. Diagnosis was achieved by histological examination of the spleen and local lymph nodes. He underwent splenectomy and did not present systemic manifestations or impaired immunity after surgery. He was diagnosed with epilepsy 10 years earlier and is currently being treated with valproic acid 300 mg twice daily. The patient was a lifelong nonsmoker without occupational or environmental exposures.

**Figure 1:**
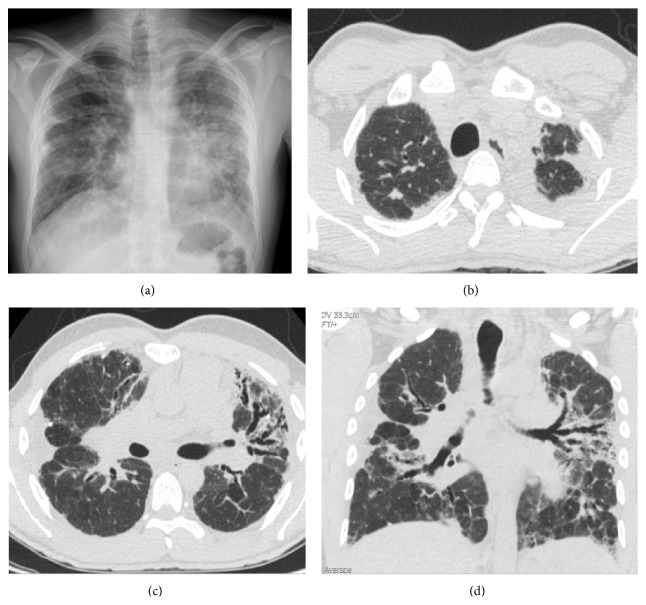
Case  1. (a) Chest radiography shows bilateral pulmonary involvement, with reticular interstitial pattern, with marked predominance in the left lung, more prominent on the left and signs of volume loss of both upper lobes with bilateral pleural thickening of apical predominance. (b) Axial high resolution computed tomography (HRCT) scan obtained through apical region shows pleural thickening, much more marked in left apex. (c) Axial HRCT scan at level of carina reveals right side marked pleural thickening and signs of pulmonary fibrosis, mainly in the left upper lobe, with the presence of multiple traction bronchiectasis, superimposed with ground-glass opacities. (d) Coronal HRCT reformatted image shows left apical and lateral right pleural thickening. Note architectural distortion reflecting fibrotic changes, accompanied by traction bronchiectasis and scattered peripheral reticular opacities.

The current process begins with a 6-month history of progressive dyspnea on exertion accompanied by fatigue, daily dry cough, and weight loss. In the previous year, the patient presented an episode of acute bronchitis and one month later he was hospitalized with pneumococcal community-acquired pneumonia with middle lobe and lingula consolidation on the chest X-Ray and positive pneumococcal antigenuria. 15 days after release from the hospital, a follow-up chest X-ray showed an interstitial pattern predominantly in the upper left lobe with bilateral pleural thickening, as well as loss of both lung volumes (Figure 1(a)). The study was complemented by HRCT which revealed left apical and right anterolateral mottled pleural thickening, predominantly in the left apex (Figure 1(b)). Signs of pulmonary fibrosis were also observed in the left upper lobe, specially lingula, with the presence of multiple traction bronchiectasis, gross distortion of the lung architecture, and ground-glass opacities (Figures 1(c) and 1(d)).

Physical examination revealed low body mass index (BMI) of 21, flattened thorax, and clubbing with basal oxygen saturation of 95%. Pulmonary auscultation revealed hypophonesis without other abnormal respiratory sounds.

The blood analysis showed normal hemogram profile and renal and hepatic function, but the study of lymphocyte subpopulations indicates high levels of B cells (CD19 +). Rheumatoid factor was 49 IU/L (normal value < 14 IU/mL) and the immunoglobulin M was 22 mg/dL (normal value: 40–230 mg/dL). Severe restrictive pattern and marked reduction of the carbon monoxide diffusing capacity (DL_CO_) were observed in pulmonary function tests (PFT). The carbon monoxide transfer coefficient (KCO) for alveolar volume was normal, whereas an increase in the ratio between the residual volume (RV) and total lung capacity (TLC) was noted (see Table 1). The analysis of the bronchoalveolar lavage (BAL) showed 57% macrophages, 28% lymphocytes, 11% neutrophils, and 1% eosinophil. BAL cellular analysis with lymphocyte subsets was CD3 80%, CD4 39%, CD 8 27%, and CD4/CD8 lymphocyte ratio of 1.4 (reference values: 1.6–1.8). The microbiological analysis of the BAL (culture for bacteria and fungi, Ziehl-Neelsen stain, silver staining, PCR mycobacteria, and Lowenstein culture) was negative.

We carried out a transbronchial biopsy using cryoprobe that showed chronic interstitial inflammatory infiltrates and areas of interstitial fibrosis. Fiber elastin stain was not performed.

**Figure 2:**
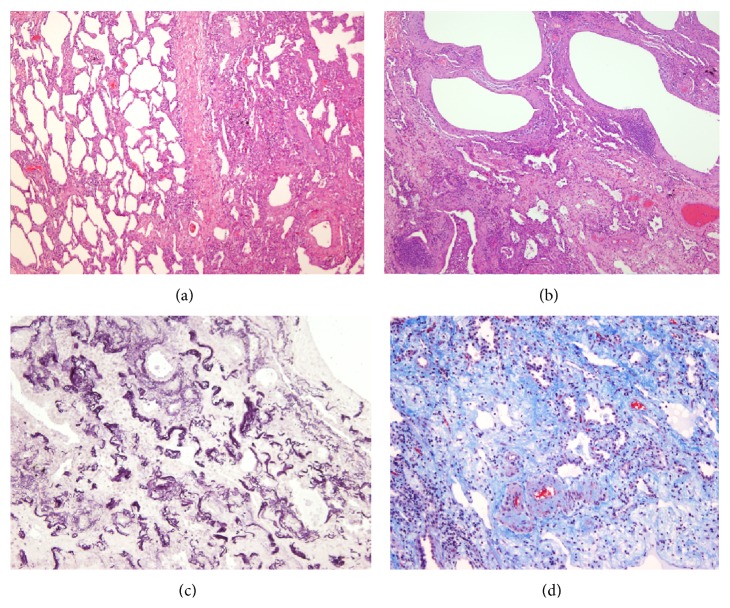
Histological features of Case  1 (surgical lung biopsy specimen). (a) Microscopic image showing sparsely cellular dense connective tissue with small vessels. Hematoxylin-eosin (HE) 100x. (b) At high power inset in area of dense fibrosis with multiple capillaries, without inflammatory infiltrate appearance. HE 250x. (c) Microscopic image shows the presence of abundant elastic fibers irregularly grouped. Orcein stain 100x. (d) Microscopic appearance of the image (b), visualized by fluorescence. Elastic fibers, which shine for eosin autofluorescence, can be observed. Fluorescence microscopy 250x.

**Figure 3:**
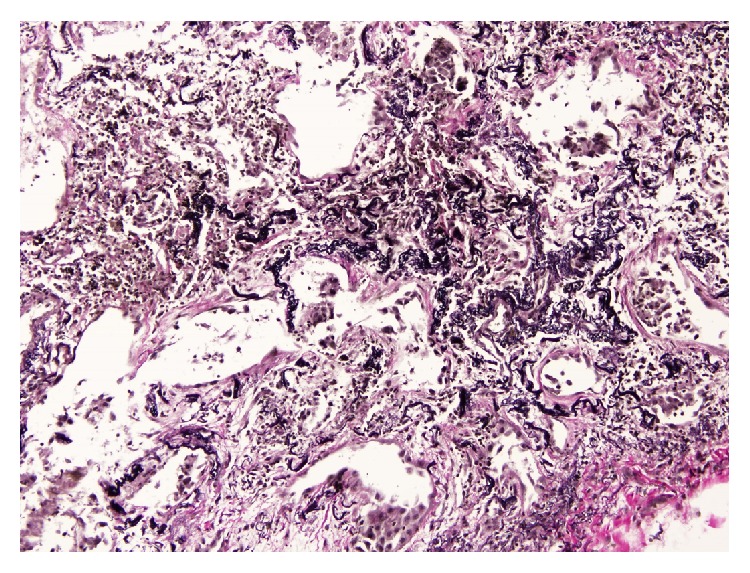
Histopathology of Case  1 (transbronchial cryobiopsy) showed abundant elastic fibers. Weigert Van Gieson stain 250x.

After 3 months, the patient developed progressive clinical deterioration with hypoxemia and oxygen desaturation during a 6-minute walk test (6 MWT) with minimal saturation of 81%. Therefore, he was treated with supplemental oxygen and oral corticosteroids (1 mg/kg/day with progressive dose reduction). SLB was performed a month after initiating the treatment with right apical pneumothorax as a complication. The biopsy revealed marked subpleural thickening and homogenous and extensive areas of fibrosis. Dense elastic fibers were seen in the subpleural area and adjacent parenchyma on elastic stain (Figure 2). After having made the diagnosis by SLB, it was decided to reexamine the histological sample from the cryobiopsy and make the Weigert Van Gieson stain, observing the elastic fibers herein (Figure 3).

**Figure 4:**
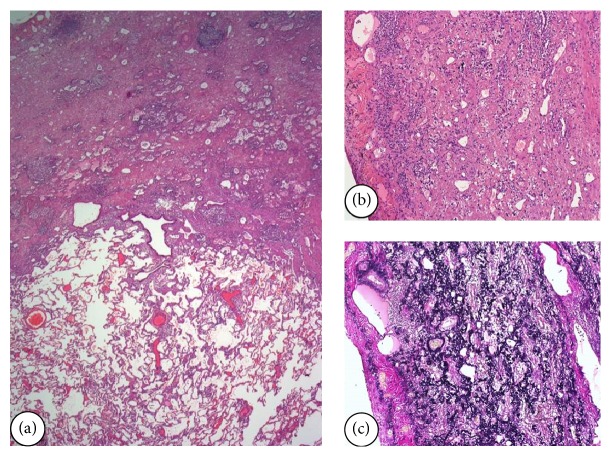
Case  1. (a) Microscopic examination of explanted lung of Case  1 (right upper lobe). The increase of elastic fibers and abrupt transition to normal parenchyma is evident. HE 200x. (b) The involved areas show collapse of alveolar spaces by interstitial material that corresponds to elastic fibers. Scant lymphocyte infiltrate. HE 400x. (c) Van Gieson stain demonstrates abnormal elastic fibers in the thickened interstitium.

The patient underwent bilateral lung transplantation (LT) at 14 months after diagnosis. Besides pleural fibroelastosis in the upper lobes (Figure 4), the pathological examination of the explant describes a pattern of nonspecific interstitial pneumonia (NSIP) in the lower lobes. Currently, the patient has 24-month survival after LT.

**Figure 5:**
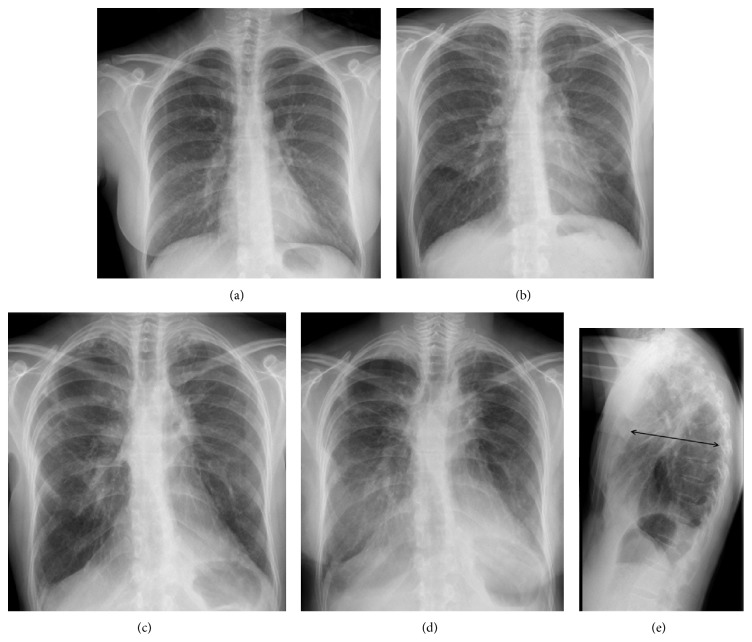
Case  2. (a) Initial chest radiograph after the diagnosis of breast cancer and before chemotherapy and radiotherapy treatment was normal. (b), (c), and (d) Chest radiographs evolution along two-year and two-month interval reveal progressive worsening. Notice progressive gradual loss of lung volume at the expense of the upper lobes, which is manifested by superior shrinkage and distortion of the pulmonary hila, as well as biapical pleural thickening. (e) Lateral chest radiograph shows flattening of the chest, observed by the decreased anteroposterior diameter of the rib cage (arrow).

*Case  2*. The patient was a 40-year-old female without history of smoking and with a history of exposure to birds. She had a history of childhood asthma and a family history of breast cancer. She was diagnosed at 37 years with bilateral invasive ductal carcinoma with axillary lymph node metastasis during pregnancy. She received neoadjuvant chemotherapy before surgery with Adriamycin and cyclophosphamide followed by docetaxel. She underwent bilateral mastectomy with dissection of axillary lymph nodes and received adjuvant chemotherapy with 4 cycles of cyclophosphamide, methotrexate, and fluorouracil with concomitant radiotherapy afterwards. The initial chest radiography after the diagnosis of breast cancer was normal. However, in the follow-up chest radiographs, a progressive loss of lung volume at the expense of the upper lobes was noticed, which manifested by superior shrinkage and distortion of the pulmonary hila, and the appearance of coarse irregular linear opacities at higher fields and biapical pleural thickening with linear opacities of scar appearance in the neighboring lung parenchyma (see Figure 5). 15 months after radiotherapy, the patient presented with acute dyspnea and chest pain. She was diagnosed with right spontaneous pneumothorax that required thorax drainage.

Subsequently, she presented exertional dyspnea with ordinary physical activity (New York Heart Association Functional Class II) and dry cough, for which she was referred to our consult for further study.

On physical examination, she had a low BMI of 19, no acropachy, and diminished global vesicular breath sound. The chest X-ray showed worsening compared to the previous one, with apical pleural thickening, hilar retraction, and flattened thorax on the lateral projection (Figure 5(e)).

The first HRCT performed to this patient 16 months after diagnosis of breast cancer showed only discrete linear opacities of fibrotic appearance in both lung apices.

**Figure 6:**
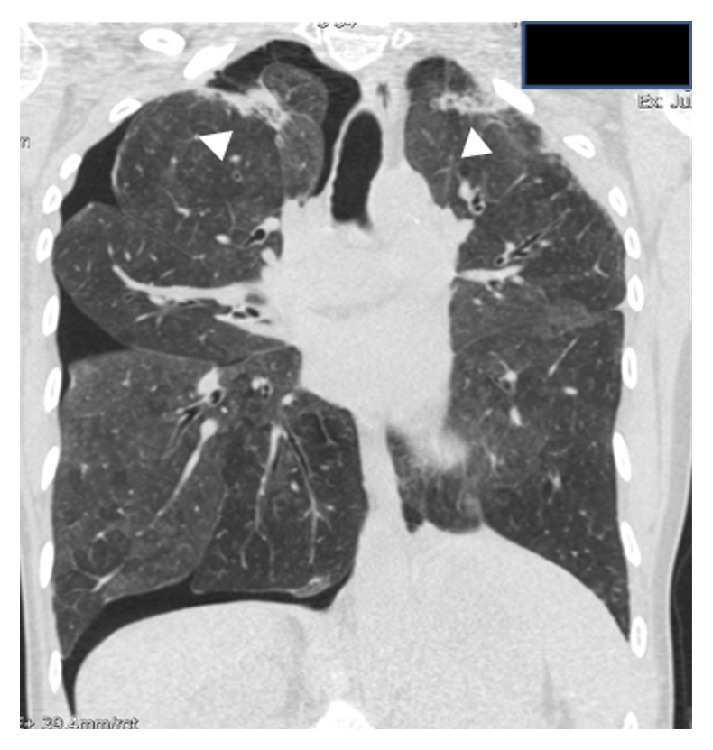
Case  2. Coronal HRCT reformatted image shows a right pneumothorax and the presence of fibrotic pulmonary infiltrates in both apical regions (arrowheads).

**Figure 7:**
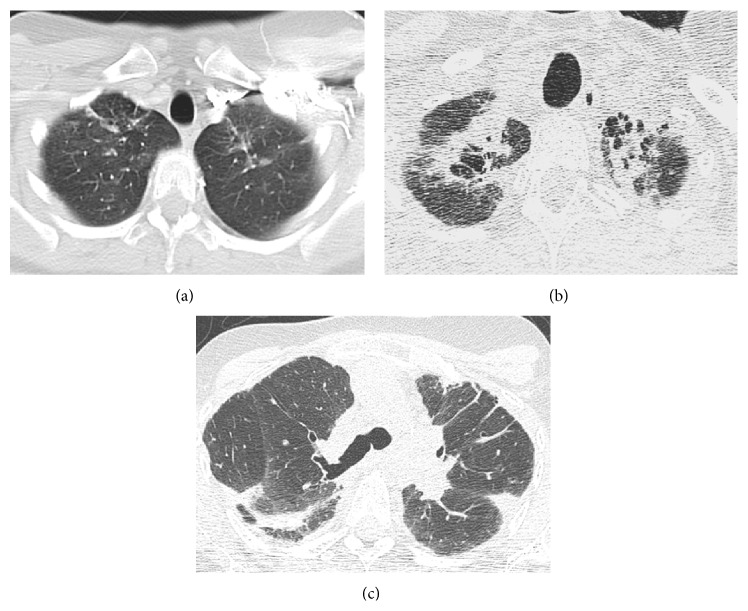
Case  2. (a) Axial HRCT scan shows very discrete lineal opacities in both lung apices. (b) Axial HRCT scan at the same level compared to (a) two years and two months later shows a loss of volume and prominent parenchymal fibrotic infiltrates. (c) Axial HRCT image at the level of carina reveals lung architectural distortion, as well as the presence of pleural thickening and subpleural parenchymal opacities.

**Figure 8:**
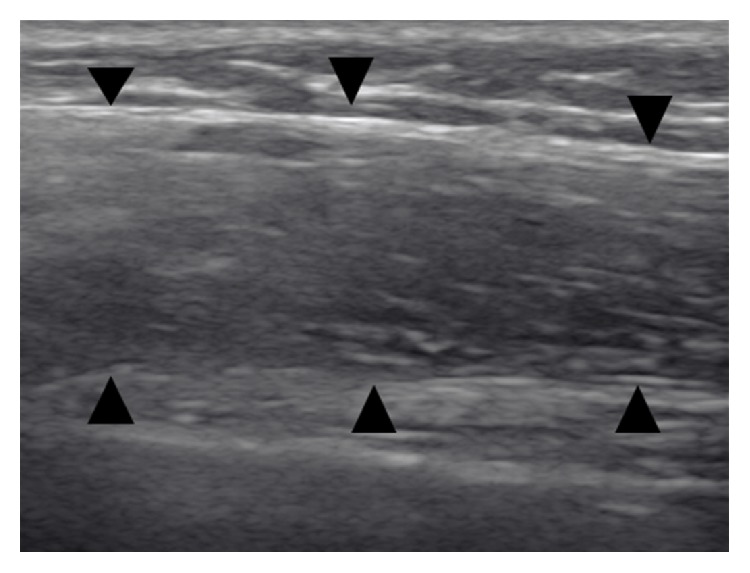
Case  2. Thoracic ultrasonography at the level of the middle third of left thorax shows a thick hypoechoic band with fine echoes, reflecting thickening of the visceral pleura (arrowheads).

However, the second HRCT, performed during the period in which the patient experienced the spontaneous pneumothorax, revealed that the upper linear opacities had progressed to pulmonary infiltrates of fibrotic appearance. Also evident was a thickening of the visceral pleura, clearly visible due to the adjacent pneumothorax (Figure 6). Finally, in a HRCT made 2 years later, further worsening of biapical infiltrates with images of honeycombing, appearance of new subpleural parenchymal consolidations, and greater lung volume loss with more architectural distortion of the parenchyma were seen (Figure 7). We performed a thoracic ultrasound (TUS) in the left hemithorax wall (lateral region from the anterior axillary line to the posterior axillary line) that showed visceral pleural thickening (Figure 8).

The BAL fluid revealed 53% macrophages, 43% lymphocytes, 2% neutrophils, and 2% eosinophils. The BAL analysis with lymphocyte subsets showed CD3 75%, CD4 16%, CD 8 60%, and CD4/CD8 lymphocyte ratio of 0.26.

**Figure 9:**
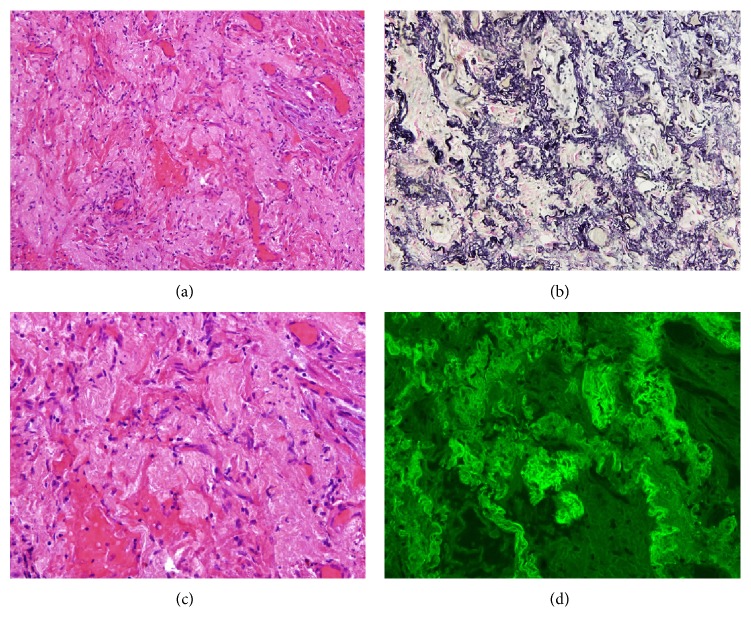
Surgical lung biopsy of the right upper lobe of Case  2. (a) The alveolar structures are completely obliterated due to accumulation of elastotic material in the wall. HE 100x. (b) At higher magnification, the presence of a very small lymphocytic infiltration is showed. (c) Using the Van Gieson stain, the elastotic content of the wall is observed and the alveolar lumen is replaced by connective tissue. (d) Using autofluorescence, the presence of numerous elastic fibbers is observed in the same field of Figure 9(b).

**Figure 10:**
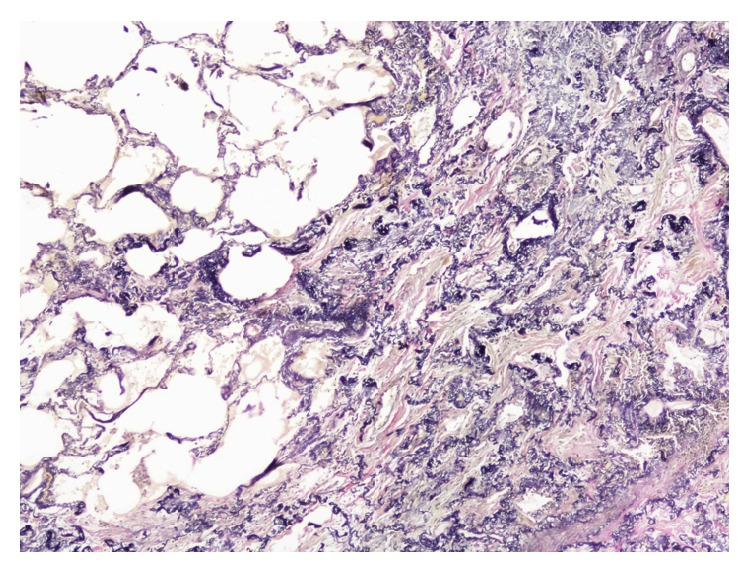
Case  2. Elastosis areas were sharply defined and showed an abrupt transition to normal tissue. Van Gieson stain.

In the blood analysis, the autoimmunity was negative and serological exams showed positive test results for* Aspergillus* and avian precipitins. PFT showed severe restrictive pattern and an increase of the relationship between residual volume (RV) and TLC (Table 1). The patient underwent breast reconstruction; therefore, SLB was delayed for seven months. Postoperatively, she presented another right loculated pneumothorax that resolved spontaneously. The pathology revealed subpleural parenchymal fibroelastosis with extension into adjacent alveolar walls predominantly in the upper lobes (Figures 9 and 10). From an oncological point of view, by not showing recurrence at 5 years after diagnosis of breast cancer, the patient was derived to the LT list and pulmonary rehabilitation with improvement of dyspnea score (NYHA class I).

## 3. Discussion

In the updated ATS/ERS classification of idiopathic interstitial pneumonias (IIPs), PPFE has been specifically introduced as rare IIPs [1, 2, 6]. The information currently available about this entity comes from case reports that have described the peculiarities of PPFE.

The cases presented here share many of the characteristics that define the disease. Both are young adults without history of smoking. The second case had a history of exposure to avian antigens and showed positive test results for* Aspergillus* and avian precipitins, consistent with other reported cases [5]. Other authors have also reported the presence of other patterns of interstitial lung disease (ILD) together with the PPFE such as usual interstitial pneumonia (UIP) and NSIP [5, 7]. This last pattern was present in the lower lobes in the explant histological description of our first case.

We cannot assure that the history of childhood's Castleman's disease of our first case could be a condition associated with the further development of PPFE. However, the history of previous respiratory infections and high titers of autoimmune markers are consistent with other series [4, 5]. On the contrary, our second patient was subjected to numerous precipitating factors such as treatment with cyclophosphamide and radiation, besides the mentioned exposure to organic antigens. The development of PPFE after the administration of these treatments presents wide variability in reported cases, ranging from 6 months to 16 years [9].

Clinically, the patients presented with the most frequently described “triad”: dyspnea, dry cough, and weight loss. Both patients were slender with low BMI (*slender stature*) and flat rib cage or abnormally narrowed anterior-posterior thoracic dimension. This last condition has been widely described in cases from Asian series [3] and has barely been described in Western patients [6]. Pneumothorax is a common manifestation and can apparently occur at any point in the natural history of the disease. In the second case, it was the first symptom of the disease. Besides, both patients had iatrogenic pneumothorax after performing SLB. The propensity of these patients to present this complication must be taken into account in the management of PPFE. The role of other diagnostic techniques such as transbronchial cryobiopsy had not been previously described in this entity. However, as evidenced by our first case, if it had been requested to perform Orcein or Van Gieson stain (that cause better visualization of the elastic fibers), the sample would have supported the diagnosis. This would have avoided the patient to undergo SLB. This is an important reason to justify knowledge of the particularities of the disease by the multidisciplinary team towards its diagnostic approach [9, 10]. The lack of initial suspicion of PPFE is a fact described in some series, leading to patients being erroneously classified of other IIPs in the first place or even having to undergo a second SLB for diagnosis [10, 11].

Chest ultrasonography or TUS is a useful tool in the diagnosis and management of many thoracic disorders, especially in pleural disease. To our knowledge, there is no information available about TUS in the assessment of PPFE. Thus, our data indicates that TUS might be a complementary imaging modality in the management of this entity and may help with the differential diagnosis of other ILDs that do not present pleural involvement.

The impairment of PFT is similar as other IIPs like idiopathic pulmonary fibrosis (IPF) with restrictive pattern and reduced DL_CO_. However, the increase of the ratio of RV/TLC is a peculiar functional impairment that is not usually seen in IPF. This finding has been described in other case reports and was also observed in our patients [12]. This particular functional feature is derived from the collapse caused by fibrosis of the upper lobes which can lead to a compensatory hyperinflation in the lower lobes [3, 12]. Currently, there is no treatment that has proven its efficacy in this disease. Our second case presented improvement in exercise tolerance with pulmonary rehabilitation, which is a nonpharmacological treatment with beneficial results in other ILDs [13]. As in IIPs, the clinical course depends in part on the time when the diagnosis is made. This is another reason to enhance the suspected diagnosis, especially in risk groups (i.e., after lung or bone marrow transplant or patients previously treated with chemotherapy). It is also important to note that, being a disease that may affect young adults, they have the option of receiving LT, as described in our cases.

In summary, a greater knowledge of the features of this disease is necessary to increase more accurate diagnosis and management by the multidisciplinary team. The latest technical modalities, such as the TUS, could help in the differential diagnosis of other ILDs. The cryobiopsy can be a useful tool in the diagnosis as it could potentially minimize the risk of pneumothorax and achieve an adequate diagnostic yield of histopathologic assessment.

## Figures and Tables

**Table 1:** Anthropometric and functional characteristics of two cases with pleuropulmonary fibroelastosis.

	Case 1	Case 2
Age (yr)	25	40
BMI (m/kg^2^)	21	19
FVC (%pred)	35	37
FEV_1_ (%pred)	44	34
FEV_1_/FVC (%)	98	75
TLC (L)	4.44	3.22
TLC (%pred)	67	61
IC (L)	1.08	0.77
IC (%pred)	32	31
RV (L)	2.45	1.92
RV (%pred)	150	113
RV/TLC (%)	227	179
IC/TLC ratio	24	23
DL_CO_ (%pred)	49	ND
KCO	88	ND
6 MWT (mt)	268	518
Resting SatO_2_	91	97
Post-6 MWT SatO_2_	81	83

BMI, body mass index; FVC, forced vital capacity; FEV_1_, forced expiratory volume in 1 s; FEV_1_/FVC, forced expiratory volume in 1 s/forced vital capacity ratio; TLC, total lung capacity; RV, residual volume; RV/TLC, residual volume/total lung capacity ratio; IC, inspiratory capacity; IC/TLC, inspiratory capacity/total lung capacity ratio; DL_CO_, diffusing capacity for carbon monoxide; KCO, carbon monoxide transfer coefficient for alveolar volume; ND: not done; 6 MWT, six-minute walk test; SatO_2_: oxygen saturation.

## References

[1] Travis W. D., Costabel U., Hansell D. M. (2013). An official American Thoracic Society/European Respiratory Society statement: update of the international multidisciplinary classification of the idiopathic interstitial pneumonias. *American Journal of Respiratory and Critical Care Medicine*.

[2] Kadoch M. A., Cham M. D., Beasley M. B. (2015). Idiopathic interstitial pneumonias: a radiology-pathology correlation based on the revised 2013 American Thoracic Society-European Respiratory Society classification system. *Current Problems in Diagnostic Radiology*.

[3] Watanabe K. (2013). Pleuroparenchymal fibroelastosis: its clinical characteristics. *Current Respiratory Medicine Reviews*.

[4] Frankel S. K., Cool C. D., Lynch D. A., Brown K. K. (2004). Idiopathic pleuroparenchymal fibroelastosis: description of a novel clinicopathologic entity. *Chest*.

[5] Reddy T. L., Tominaga M., Hansell D. M. (2012). Pleuroparenchymal fibroelastosis: a spectrum of histopathological and imaging phenotypes. *European Respiratory Journal*.

[6] Beynat-Mouterde C., Beltramo G., Lezmi G. (2014). Pleuroparenchymal fibroelastosis as a late complication of chemotherapy agents. *European Respiratory Journal*.

[7] Ofek E., Sato M., Saito T. (2013). Restrictive allograft syndrome post lung transplantation is characterized by pleuroparenchymal fibroelastosis. *Modern Pathology*.

[8] von der Thüsen J. H., Hansell D. M., Tominaga M. (2011). Pleuroparenchymal fibroelastosis in patients with pulmonary disease secondary to bone marrow transplantation. *Modern Pathology*.

[9] Camus P., von der Thüsen J., Hansell D. M., Colby T. V. (2014). Pleuroparenchymal fibroelastosis: one more walk on the wild side of drugs?. *European Respiratory Journal*.

[10] Noh H. J., Seo Y., Huo S. M., Kim T. J., Kim H. L., Song J. S. (2014). Idiopathic pleuroparenchymal fibroelastosis presenting in recurrent pneumothorax: a case report. *Tuberculosis and Respiratory Diseases*.

[11] Hirota T., Yoshida Y., Kitasato Y. (2015). Histological evolution of pleuroparenchymal fibroelastosis. *Histopathology*.

[12] Kusagaya H., Nakamura Y., Kono M. (2012). Idiopathic pleuroparenchymal fibroelastosis: consideration of a clinicopathological entity in a series of Japanese patients. *BMC Pulmonary Medicine*.

[13] Kenn K., Gloeckl R., Behr J. (2013). Pulmonary rehabilitation in patients with idiopathic pulmonary fibrosis—a review. *Respiration*.

